# Preoperative paraspinous muscle sarcopenia and physical performance as prognostic indicators in non‐small‐cell lung cancer

**DOI:** 10.1002/jcsm.12691

**Published:** 2021-03-04

**Authors:** Shinya Tanaka, Naoki Ozeki, Yota Mizuno, Hiroki Nakajima, Keiko Hattori, Takayuki Inoue, Motoki Nagaya, Takayuki Fukui, Shota Nakamura, Masaki Goto, Tomoshi Sugiyama, Yoshihiro Nishida, Toyofumi Fengshi Chen‐Yoshikawa

**Affiliations:** ^1^ Department of Rehabilitation Nagoya University Hospital Nagoya Japan; ^2^ Department of Thoracic Surgery Nagoya University Graduate School of Medicine Nagoya Japan; ^3^ Department of Orthopaedic Surgery Nagoya University Graduate School and School of Medicine Nagoya Japan

**Keywords:** Sarcopenia, Physical performance, Lung neoplasm, Thoracic surgical procedures, Post‐operative outcomes

## Abstract

**Background:**

Despite the associations of both preoperative sarcopenia and physical performance with post‐operative mortality in non‐small‐cell lung cancer (NSCLC), there have been no comprehensive studies of the impact of physical status on prognosis. This study was performed to investigate the prognostic significance of preoperative sarcopenia and physical performance in NSCLC.

**Methods:**

This retrospective cohort study was performed in NSCLS patients undergoing curative lung resection at a university hospital between January 2014 and December 2017. The patients were divided into four groups according to the skeletal muscle index [sarcopenia (lowest sex‐specific tertile) and non‐sarcopenia] and 6 min walking distance (6MWD) [short distance (<400 m) and long distance (≥400 m)]. Sarcopenia was assessed by preoperative cross‐sectional areas of right and left paraspinous muscles at the level of the 12th thoracic vertebra from computed tomography images, and physical performance was determined by preoperative 6MWD. The primary and secondary endpoints were post‐operative overall survival (OS) and disease‐free survival (DFS).

**Results:**

The 587 patients [mean age: 68.5 ± 8.8 years, 399 men (68%)] included in the study were divided into the non‐sarcopenia/long‐distance group (58%), sarcopenia/long‐distance group (26%), non‐sarcopenia/short‐distance group (9%), and sarcopenia/short‐distance group (7%). A total of 109 (18.6%) deaths and 209 (35.6%) combined endpoints were observed over a mean follow‐up of 3.1 ± 1.3 years. After adjusting for other covariates, the sarcopenia/short‐distance group showed significant associations with shorter OS (hazard ratio, 3.38; 95% confidence interval, 1.79–6.37; *P* < 0.001) and DFS (hazard ratio, 2.11; 95% confidence, 1.27–3.51; *P* = 0.004) compared with the non‐sarcopenia/long‐distance group on multivariate analyses. Although not significant, adding skeletal muscle index and 6MWD to the pre‐existing risk model increased the area under the curve on time‐dependent receiver operating characteristic curve analysis for OS and DFS, except within 2 years of surgery.

**Conclusions:**

The presence of both preoperative paraspinous muscle sarcopenia and short distance in 6MWD had an adverse effect on post‐operative prognosis in patients with NSCLC, suggesting that preoperative assessment of thoracic sarcopenia and physical performance may be useful for risk stratification of surgical candidates with potential for targeted interventions.

## Introduction

Lung cancer is one of the main causes of mortality around the world and has been reported to account for 1.7 million deaths every year.^1^ Approximately 80% of cases of primary lung cancer are classified as non‐small‐cell lung cancer (NSCLC), for which the standard treatment of choice in early stage disease is surgical resection.^2^ The outcome following resection of NSCLC remains poor in a proportion of patients despite improvements in survival rates due to progress in the development of diagnostic methods and treatment strategies,^3^ which may be related to cancer‐specific factors and differences in physical status between individuals.^4^ With ageing of the population, it is necessary to develop means for accurately evaluating the preoperative state of patients and to predict prognosis and physical performance following surgery.^5^, ^6^

Sarcopenia, defined as a progressive and generalized skeletal muscle disorder characterized by loss of muscle mass and strength, is common among older adults but can also occur in younger individuals.^7^ Sarcopenia has been shown to be associated with frailty, disability, falls, and poor prognosis,^7^ and the presence of sarcopenia prior to surgery is a strong prognostic factor for mortality in NSCLC patients.^8^, ^9^ Assessment of the psoas muscle at the level of the third lumbar vertebra (L3) on single‐slice computed tomography (CT) is often used as an objective means of identifying sarcopenia.^8^, ^10^ However, the standard imaging modality in preoperative evaluation of NSCLC, chest CT, does not routinely visualize the L3 level, and supplementary abdominal CT would result in both additional medical costs and radiation exposure to the patient. There is increasing interest in measurement of skeletal muscle mass at the level of the 12th thoracic vertebra (T12) on chest CT, and sarcopenia in the thoracic area was shown to be correlated to poorer survival in various patient populations, including those with NSCLC.^11^, ^12^, ^13^, ^14^ In addition, measurement of physical performance has been recommended for assessment of the severity of sarcopenia, and severe sarcopenia was shown to be closely related to both frailty and cachexia.^7^, ^15^ Physical performance is frequently assessed in NSCLC patients based on the 6 min walking distance (6MWD),^16^ and preoperative poor physical performance defined according to short 6MWD has been reported to be useful for predicting outcomes.^17^, ^18^ Taken together, these observations suggest the importance of comprehensive assessment of preoperative physical state for risk stratification in cases of NSCLC. However, there have been no previous reports of the comprehensive assessment of overall physical status, including sarcopenia and physical performance, and its impact on prognosis in NSCLC.

The present study was performed to investigate the prognostic significance of preoperative thoracic sarcopenia and physical performance status in patients with NSCLC.

## Materials and methods

### Study population

This retrospective review was performed among consecutive NSCLS patients undergoing curative lung resection at Nagoya University Hospital, in whom 6MWD was evaluated preoperatively between January 2014 and December 2017. Patients with non‐curative (R1 or R2) resection and pathological stage IV disease were excluded. The study was approved by the Institutional Review Board of Nagoya University Hospital and was performed in accordance with the tenets of the Declaration of Helsinki and the Japanese Ethical Guidelines for Medical and Health Research Involving Human Subjects.

### Data collection and follow‐up

The clinical details of presentation and demographic and biochemical data were obtained from electronic medical records. The pathological stage was determined according to the eighth edition of the tumour–node–metastasis staging system for lung cancer, and the tumour grades were classified according to the World Health Organization classification of histological differentiation.^19^, ^20^

Patients were followed up post‐operatively by thoracic surgeons and/or respirologists with routine assessments including physical examination, chest radiography, and blood analyses. Further evaluations, including chest and abdominal CT, brain magnetic resonance imaging (MRI), and bone scintigraphy, were performed at the discretion of the attending physician. Recurrence was diagnosed based on physical examination and diagnostic imaging of the lesions, and diagnosis was confirmed histologically where feasible. The date of cancer recurrence was defined as the date of histological confirmation or the date of identification based on clinicoradiological findings by a physician. All patients were prospectively followed up, and data on cancer recurrence and survival status were obtained from annual medical records reviews or mail‐based surveys. The post‐operative complications recorded within 30 days after surgery were registered in accordance with the predefined definitions of the National Clinical Database.^21^ There were six respiratory complications: pneumonia, empyema, bronchial fistula, respiratory dysfunction, acute interstitial pneumonia, and atelectasis.

Overall survival (OS) and disease‐free survival (DFS) were the primary and secondary endpoints of the study, respectively. The time to the endpoint was calculated as the number of days from the date of surgery to the date of the event. In addition, secondary outcomes included post‐operative complications and length of hospital stay.

### Computed tomography and physical performance evaluation

Preoperative CT was performed within 2 months prior to surgery. The sum of cross‐sectional areas of the right and left paraspinous muscles (i.e. the iliocostalis, longissimus, and spinalis) was determined on preoperative chest CT at the T12 level to assess preoperative thoracic sarcopenia.^11^, ^13^, ^14^ Briefly, the paraspinous muscle area was defined semi‐automatically using SYNAPSE VINCENT™ software (Fujifilm Medical Co., Ltd., Tokyo, Japan), and the muscle area was quantified based on thresholds of −29 to +150 Hounsfield units. To ensure lack of bias in measurements and calculations, image analysis was performed by an investigator blinded to the survival information of the patients. To normalize muscle area for height, the skeletal muscle index (SMI) was calculated by dividing the total muscle cross‐sectional area (cm^2^) by the square of the patient's height (m^2^). Patients were stratified separately by tertiles according to the normalized SMI for male and female, and sarcopenia was defined as the presence of normalized SMI in the lowest sex‐specific tertile.

Six minute walking distance was measured to assess physical performance within 7 days prior to surgery according to standard guidelines established by the American Thoracic Society.^22^ Patients received standardized instructions to cover as much distance as possible within a period of 6 min, and the use of assistive devices was permitted if necessary, and were divided into the short‐distance group (6MWD < 400 m) and long‐distance group (6MWD ≥ 400 m).^18^, ^23^

### Statistical analysis

Normally distributed continuous variables are expressed as the mean ± standard deviation, while non‐normally distributed variables are presented as the median and inter‐quartile range. Categorical variables are expressed as numbers and percentages. The cohort was divided into four groups: (i) non‐sarcopenia/long‐distance group; (ii) sarcopenia/long‐distance group; (iii) non‐sarcopenia/short‐distance group; and (iv) sarcopenia/short‐distance group. Differences between groups were evaluated by one‐way analysis of variance or the Kruskal–Wallis test for continuous variables, and *χ* ^2^ or Fisher's exact test for dichotomous variables, as appropriate.

Event‐free survival curves were plotted using the Kaplan–Meier survival method and were compared with log‐rank statistics. Age, sex, body mass index (BMI), smoking status, pathological stage, serum albumin level, extent of resection, forced expiratory volume in 1 s, and the lung diffusion capacity for carbon monoxide (DLco) status were selected as adjustment variables in multivariate Cox regression analyses for OS and DFS as outcomes based on their clinical importance and the results of previous studies. To evaluate whether the prognostic impacts of sarcopenia and 6MWD are influenced by adjustment variables, we examined the interactions for both prognostic outcomes in multivariate Cox regression analyses. In addition, as there was no established cut‐off value of SMI for sarcopenia, we conducted sensitivity analysis for various cut‐off values for SMI (lowest sex‐specific quartile and Kaplan's value). Hazard ratios (HRs) are reported with the corresponding 95% confidence intervals (CIs).

To evaluate the incremental prognostic value of preoperative thoracic sarcopenia and physical performance, time‐dependent receiver operating characteristic (ROC) curves for OS and DFS were used to compare the accuracy of adding both SMI and 6MWD to the baseline model incorporating pre‐existing risk factors that included all variables used for adjustment. The areas under the ROC curve (AUCs) were compared by using the R package ‘timeROC’.^24^

Multiple imputation was used to take into account the missing covariate data to construct multivariable Cox regression models. We created 20 datasets using a chained‐equations procedure.^25^ Parameter estimates were obtained for each dataset and subsequently combined to produce an integrated result using the method described by Barnard and Rubin.^26^

In all analyses, a two‐tailed *P* < 0.05 was taken to indicate statistical significance, and Bonferroni correction was applied for multiple comparisons. Statistical analyses were performed using SPSS Version 23.0 (IBM Corporation, Armonk, NY) and R Version 3.2.1 (R Foundation for Statistical Computing, Vienna, Austria).

## Results

**Figure 1:**
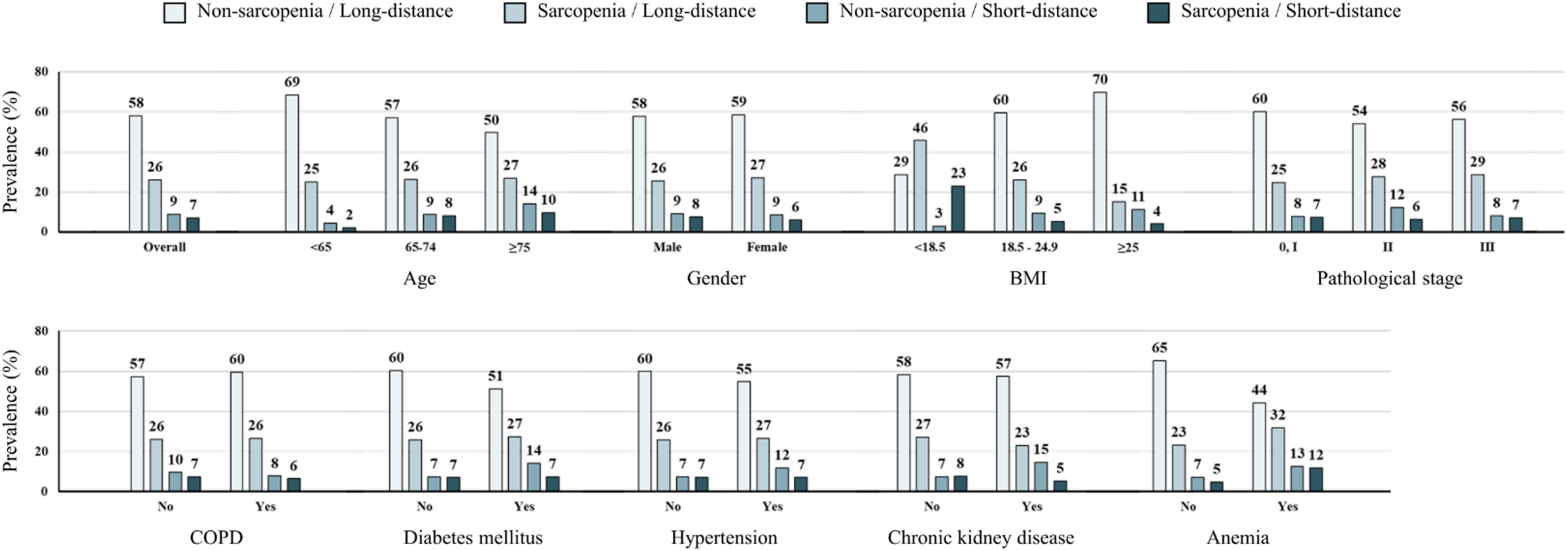
Prevalence of sarcopenia and low physical performance. Sarcopenia was defined as normalized skeletal muscle index in the lowest sex‐specific tertile. Short‐distance was defined as 6 min walking distance <400 m. BMI, body mass index; COPD, chronic obstructive pulmonary disease.

A total of 657 NSCLC patients were initially enrolled in the study, and 70 of these patients were excluded (non‐curative resection, *n* = 60; Stage IV, *n* = 9; and CT imaging unavailable, *n* = 1). Consequently, 587 patients (399 men, 68%) with a mean age of 68.5 ± 8.8 years were included in the analyses. The mean SMI was 12.1 ± 2.6 cm^2^/m^2^ for men and 10.7 ± 2.1 cm^2^/m^2^ for women, and the mean 6MWD for the total study population was 489 ± 109 m. Male and female patients with normalized SMI < 11.0 and <9.84 cm^2^/m^2^, respectively, were included in the lowest tertile and were classified as having sarcopenia. Analyses were performed in subgroups divided according to preoperative SMI and 6MWD as well as baseline characteristics (Supporting Information, *Figure* S1). SMI of male patients and 6MWD of the total patient population showed inverse associations with age, but SMI was not significantly related to age in female patients. There were no significant differences in 6MWD according to sex or in SMI and 6MWD according to pathological stage. BMI < 18.5 kg/m^2^ was associated with greater likelihoods of positivity for both sarcopenia and short‐distance 6MWD (*Figure* 1).

The baseline characteristics of the total patient population as well as subgroups stratified according to preoperative sarcopenic status and 6MWD are shown in *Table* 1. About half of the patients underwent video‐assisted thoracoscopic surgery, 485 (82.6%) patients had lobectomy (including 18 with pneumonectomy), and 4.1% and 8.3% received neoadjuvant and adjuvant therapy, respectively. The study population was divided into the non‐sarcopenia/long‐distance group (341 patients, 58%), sarcopenia/long‐distance group (153 patients, 26%), non‐sarcopenia/short‐distance group (52 patients, 9%), and sarcopenia/short‐distance group (41 patients, 7%). The sarcopenia/short‐distance group had significantly greater age, lower BMI, higher prevalence of peripheral vascular disease, lower albumin and haemoglobin levels, and higher total neutrophil count than the non‐sarcopenia/long‐distance group. The sarcopenia/short‐distance group also had significantly lower pulmonary function excluding forced expiratory volume in the first second to forced vital capacity ratio, lower SMI, and shorter 6MWD than the non‐sarcopenia/long‐distance group. There were no significant differences in sex, smoking status, pathological stage, histological type, or surgery‐related factors between the groups. Similar results were obtained in analyses of subgroups of patients divided according to preoperative sarcopenic status and 6MWD (*Table* S1). There were no significant differences in overall complications among groups, and the incidence of respiratory complications was higher in the sarcopenia/short‐distance group although the results of pairwise comparisons were not significant (*Table* 2). The sarcopenia/short‐distance group had significantly longer post‐operative hospital stay than the non‐sarcopenia/long‐distance group.

**Table 1:** Baseline patient characteristics

	Overall (*n* = 587)	Non‐sarcopenia/long‐distance (*n* = 341; 58%)	Sarcopenia/long‐distance (*n* = 153; 26%)	Non‐sarcopenia/short‐distance (*n* = 52; 9%)	Sarcopenia/short‐distance (*n* = 41; 7%)	*P* value
Age, mean ± SD, years	68.5 ± 8.8	67.8 ± 8.4	68.2 ± 9.8	70.5 ± 8.7	72.2 ± 6.5^a^	0.006
Age group, *n* (%)						
<65	140 (23.9)	96 (28.2)	35 (22.9)	6 (11.5)	3 (7.3)	
65–74	312 (53.2)	178 (52.2)	82 (53.6)	27 (51.9)	25 (61.0)	0.005
≥75	135 (23.0)	67 (19.6)	36 (23.5)	19 (36.5)^a^	13 (31.7)^a^	
Male, *n* (%)	399 (68.0)	231 (67.7)	102 (66.7)	36 (69.2)	30 (73.2)	0.879
Height, mean ± SD, cm	161.1 ± 8.6	161.4 ± 8.6	161.5 ± 8.5	159.2 ± 8.8	159.6 ± 8.8	0.212
Body weight, mean ± SD, kg	58.4 ± 10.8	60.4 ± 10.6	54.9 ± 10.1^a^	59.4 ± 10.0^b^	52.7 ± 10.2^a^ ^,^ ^c^	<0.001
BMI, mean ± SD, kg/m^2^	22.4 ± 3.3	23.1 ± 3.1	21.0 ± 3.1^a^	23.3 ± 3.0^b^	20.7 ± 3.7^a^ ^,^ ^c^	<0.001
BMI group, *n* (%)						
<18.5	70 (11.9)	20 (5.9)	32 (20.9)	2 (3.6)	16 (39.0)	
18.5–24.9	391 (66.6)	233 (68.3)	102 (66.7)	36 (69.2)	20 (48.8)	<0.001
≥25	126 (21.5)	88 (25.8)	19 (12.4)^a^	14 (26.9)^b^	5 (12.2)^a^ ^,^ ^c^	
Smoking status, *n* (%)						
Never smoker	168 (28.6)	100 (29.3)	48 (31.4)	13 (25.0)	7 (17.1)	0.301
Ever smoker (current or ex‐smoker)	419 (71.4)	241 (70.7)	105 (68.6)	39 (75.0)	34 (82.9)	
Co‐morbidities, *n* (%)						
COPD	220 (37.5)	131 (38.4)	58 (37.9)	17 (32.7)	14 (34.1)	0.839
Diabetes	135 (23.0)	69 (20.2)	37 (24.2)	19 (36.5)^a^	10 (24.4)	0.071
Hypertension	203 (34.6)	111 (32.6)	54 (35.3)	24 (46.2)	14 (34.1)	0.291
Chronic kidney disease	131 (22.3)	75 (22.0)	30 (19.6)	19 (36.5)	7 (17.1)	0.061
Anaemia	199 (33.9)	88 (25.8)	63 (41.2)^a^	25 (48.1)^a^	23 (56.1)^a^	<0.001
Peripheral vascular disease	25 (4.3)	6 (1.8)	2 (1.3)	8 (15.4)^a^ ^,^ ^b^	9 (22.0)^a^ ^,^ ^b^	<0.001
Cerebrovascular disease	38 (6.5)	17 (5.0)	10 (6.5)	9 (17.3)^a^	2 (4.9)	0.009
Laboratory findings						
Albumin, mean ± SD, g/dL	4.0 ± 0.4	4.0 ± 0.4	4.0 ± 0.4	4.0 ± 0.4	3.8 ± 0.6^a^ ^,^ ^b^	<0.001
Haemoglobin, mean ± SD, g/dL	13.1 ± 1.6	13.3 ± 1.5	12.7 ± 1.6^a^	12.6 ± 1.3^a^	12.5 ± 2.0^a^	<0.001
Creatinine, mean ± SD, mg/dL	0.91 ± 0.94	0.88 ± 0.86	0.85 ± 0.71	1.32 ± 1.81^a^ ^,^ ^b^	0.84 ± 0.57	0.011
eGFR, mean ± SD, mL/min/1.73 m^2^	72.6 ± 21.8	71.3 ± 17.3	75.7 ± 22.2	66.6 ± 27.3	80.4 ± 37.6^c^	0.003
CRP, median (IQR), mg/L	0.08 [0.04–0.25]	0.07 [0.03–0.23]	0.07 [0.03–0.28]	0.15 [0.07–0.28]	0.12 [0.08–0.27]	0.014
TLC, mean ± SD, cells/mm^3^	1710 ± 614	1750 ± 636	1652 ± 573	1685 ± 591	1639 ± 599	0.328
TNC, mean ± SD, cells/mm^3^	4370 ± 2156	4249 ± 1534	4418 ± 2113	4190 ± 1656	5441 ± 5126^a^ ^–^ ^c^	0.008
NLR, mean ± SD	2.9 ± 2.1	2.8 ± 2.0	3.0 ± 2.3	2.9 ± 1.7	3.7 ± 2.7	0.097
Pathological stage, *n* (%)						
0, I	344 (58.6)	207 (60.7)	85 (55.6)	27 (51.9)	25 (61.0)	
II	131 (22.3)	71 (20.8)	36 (23.5)	16 (30.8)	8 (19.5)	0.729
III	112 (19.1)	63 (18.5)	32 (20.9)	9 (17.3)	8 (19.5)	
Histological type, *n* (%)						
Squamous cell carcinoma	167 (28.4)	88 (25.8)	46 (30.1)	19 (36.5)	14 (34.1)	
Adenocarcinoma	393 (67.0)	235 (68.9)	102 (66.7)	31 (59.6)	25 (61.0)	0.612
Others	27 (4.6)	18 (5.3)	5 (3.3)	2 (3.8)	2 (4.9)	
Operation time, median (IQR), min	150 [120–187]	150 [116–187]	159 [130–188]	148 [122–169]	133 [115–192]	0.222
Blood loss during surgery, median (IQR), g	52 [19–140]	45 [17–123]	66 [21–187]	61 [15–178]	61 [26–184]	0.253
Surgical approach, *n* (%)						0.377
Open	258 (44.0)	142 (41.6)	74 (48.4)	21 (40.4)	21 (51.2)	
VATS	329 (56.0)	199 (58.4)	79 (51.6)	31 (59.6)	20 (48.8)	
Extent of resection, *n* (%)						0.337
Lobectomy	485 (82.6)	276 (80.9)	132 (86.3)	41 (78.8)	36 (87.8)	
Sublobar resection	102 (17.4)	65 (19.1)	21 (13.7)	11 (21.2)	5 (12.2)	
Neoadjuvant therapy, *n* (%)	24 (4.1)	14 (4.1)	6 (3.9)	3 (5.8)	1 (2.4)	0.880
Adjuvant therapy, *n* (%)	49 (8.3)	33 (9.7)	10 (6.5)	5 (9.6)	1 (2.4)	0.331
Preoperative pulmonary function test						
VC, mean ± SD, L	3.3 ± 0.8	3.4 ± 0.8	3.2 ± 0.7^a^	3.1 ± 0.7^a^	2.7 ± 0.7^a^ ^,^ ^b^	<0.001
VC, mean ± SD, %predicted	102.0 ± 16.2	104.8 ± 14.6	99.9 ± 16.6^a^	100.2 ± 16.8	89.1 ± 18.6^a^ ^–^ ^c^	<0.001
FEV_1_, mean ± SD, L	2.3 ± 0.6	2.4 ± 0.6	2.3 ± 0.6	2.1 ± 0.6	1.9 ± 0.5^a^ ^,^ ^b^	<0.001
FEV_1_, mean ± SD, %predicted	92.4 ± 19.3	93.9 ± 17.9	92.2 ± 20.9	91.2 ± 21.3	82.9 ± 19.6^a^ ^,^ ^b^	0.007
FEV_1_/FVC, mean ± SD	70.0 ± 10.1	69.4 ± 8.8	71.2 ± 11.2	69.7 ± 11.9	71.3 ± 12.7	0.241
DLco, mean ± SD, %predicted	103.1 ± 25.9	105.3 ± 24.6	103.2 ± 26.2	95.3 ± 27.3	93.7 ± 30.4^a^	0.006
SMI, mean ± SD, cm^2^/m^2^						
Male	12.1 ± 2.6	13.5 ± 1.8	9.5 ± 1.6^a^	12.9 ± 1.4^b^	9.1 ± 1.7^a^ ^,^ ^c^	<0.001
Female	10.7 ± 2.1	11.7 ± 1.5	8.8 ± 1.2^a^	11.9 ± 1.5^b^	7.8 ± 1.8^a^ ^,^ ^c^	<0.001
6MWD, mean ± SD, m	489 ± 109	526 ± 74	516 ± 75	323 ± 70^a^ ^,^ ^b^	292 ± 106^a^ ^,^ ^b^	<0.001

6MWD, 6 min walking distance; BMI, body mass index; COPD, chronic obstructive pulmonary disease; CRP, C‐reactive protein; DLco, lung diffusion capacity for carbon monoxide; eGFR, estimated glomerular filtration rate; FEV_1_, forced expiratory volume in 1 s; FVC, forced vital capacity; IQR, inter‐quartile range; NLR, neutrophil–lymphocyte ratio; SD, standard deviation; SMI, skeletal muscle index; TLC, total lymphocyte count; TNC, total neutrophil count; VATS, video‐assisted thoracoscopic surgery; VC, vital capacity.

Sarcopenia was defined as normalized SMI in the lowest sex‐specific tertile. Short‐distance was defined as 6MWD < 400 m.

^a^ Significant difference from non‐sarcopenia/long‐distance group.

^b^ Significant difference from sarcopenia/long‐distance group.

^c^ Significant difference from non‐sarcopenia/short‐distance group.

**Table 2:** Post‐operative short‐term outcomes

	Overall (*n* = 587)	Non‐sarcopenia/long‐distance (*n* = 341; 58%)	Sarcopenia/long‐distance (*n* = 153; 26%)	Non‐sarcopenia/short‐distance (*n* = 52; 9%)	Sarcopenia/short‐distance (*n* = 41; 7%)	*P* value
Any kind of complications, *n* (%)	115 (19.6)	61 (17.9)	31 (20.3)	11 (21.2)	12 (29.3)	0.363
Respiratory complications, *n* (%)	47 (8.0)	25 (7.3)	12 (7.8)	2 (3.8)	8 (19.5)	0.032
Length of hospital stay, median (IQR), days	6 [5–8]	6 [5–7]	6 [5–8]	6 [5–7]	7 [6–10]^a^	0.002

IQR, inter‐quartile range.

Respiratory complications included six complications; pneumonia, empyema, bronchial fistula, respiratory dysfunction, acute interstitial pneumonia, and atelectasis.

^a^ Significant difference from non‐sarcopenia/long‐distance group.

**Figure 2:**
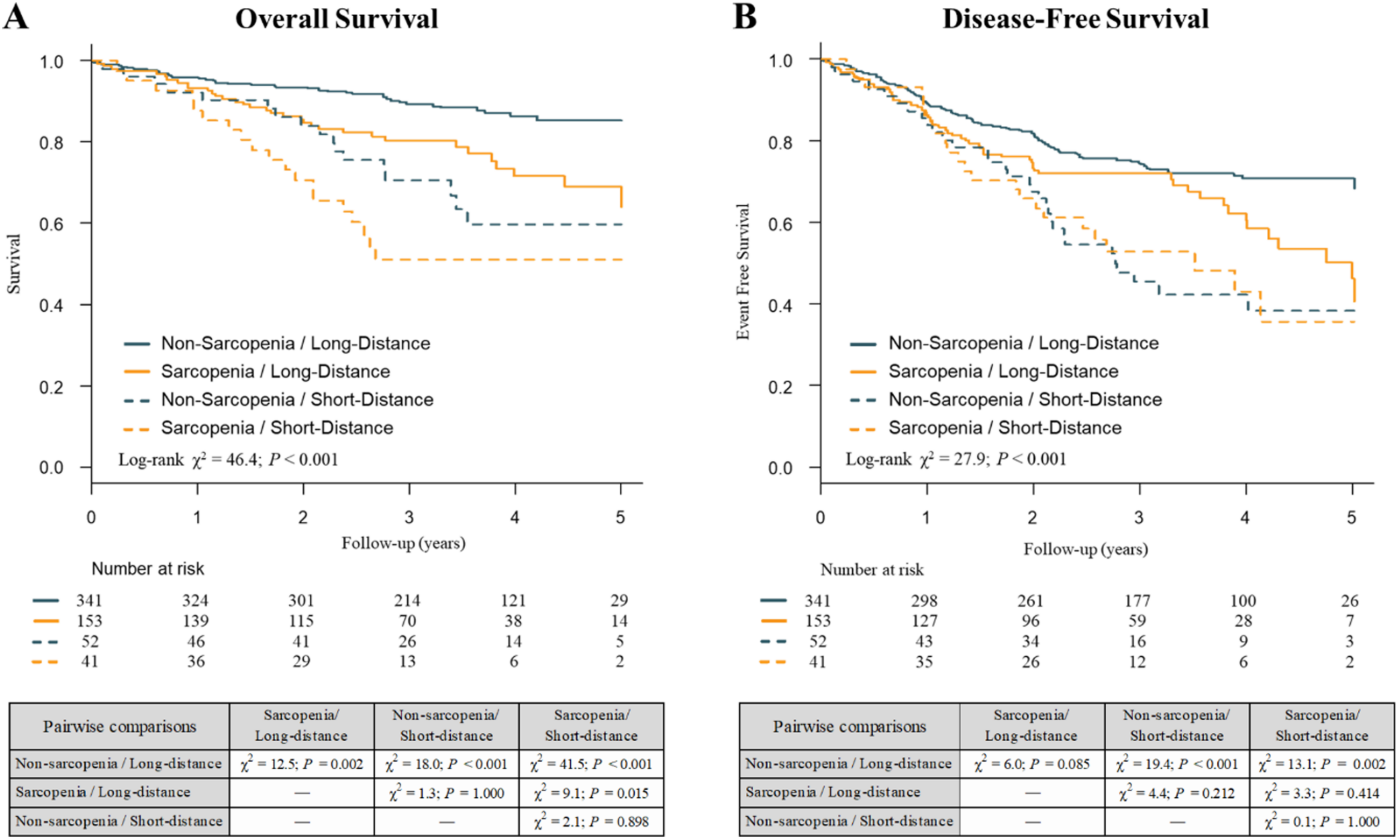
Kaplan–Meier curves for *(A)* overall survival and *(B)* disease‐free survival according to sarcopenic status and physical performance. Sarcopenia was defined as normalized skeletal muscle index in the lowest sex‐specific tertile. Short distance was defined as 6 min walking distance <400 m.

Over a mean follow‐up period of 3.1 ± 1.3 years, a total of 109 (18.6%) deaths and 209 (35.6%) combined endpoints were observed, and Kaplan–Meier curves indicated that sarcopenia and/or short‐distance 6MWD were associated with shorter OS and DFS (*Figure* 2). The results of Cox regression analyses for OS and DFS are shown in *Table* 3. After adjusting for possible confounding factors, the risk of mortality was higher in patients with sarcopenia and/or short‐distance 6MWD than in those without either indicator (i.e. the non‐sarcopenia/long‐distance group), and this increased risk varied according to the indicators (sarcopenia/long‐distance group: HR, 1.78; 95% CI, 1.08–2.93; *P* = 0.024; non‐sarcopenia/short‐distance group: HR, 2.26; 95% CI, 1.22–4.19; *P* = 0.010; and sarcopenia/short‐distance group: HR, 3.38; 95% CI, 1.79–6.37; *P* < 0.001). In the adjusted models, short‐distance 6MWD was significantly associated with shorter DFS regardless of the presence or absence of sarcopenia (non‐sarcopenia/short‐distance group: HR, 2.09; 95% CI, 1.36–3.21; *P* < 0.001; sarcopenia/short‐distance group: HR, 2.11; 95% CI, 1.27–3.51; *P* = 0.004). There were significant interactions with extent of resection (*P* for interaction <0.001 for OS and 0.007 for DFS), and the prognostic impacts of sarcopenia and/or short‐distance 6MWD were significantly higher in the sublobar resection group compared with the lobectomy group. No other significant interactions were observed. After adjusting for possible confounding factors, the SMI was significantly inversely associated with OS in the total population (adjusted HR for each 1 cm^2^/m^2^ decrease in SMI: 1.09; 95% CI: 1.01–1.18; *P* = 0.025), and 6MWD was significantly inversely correlated with both OS (adjusted HR for each 50 m decrease in 6MWD: 1.14; 95% CI: 1.05–1.25; *P* = 0.003) and DFS (adjusted HR for each 50 m decrease in 6MWD: 1.09; 95% CI: 1.02–1.17; *P* = 0.008) (*Table* S2). Sensitivity analysis using the lowest sex‐specific quartile and Kaplan's value as cut‐off values for SMI yielded similar results (*Table* S3).

**Table 3:** Unadjusted and adjusted Cox regression analyses for post‐operative outcomes

Variable	Univariate analysis			Multivariate analysis		
	HR	95% CI	*P* value	HR	95% CI	*P* value
Overall survival						
Indicator						
Non‐sarcopenia/long‐distance	1.00	[Reference]		1.00	[Reference]	
Sarcopenia/long‐distance	2.27	1.43–3.60	<0.001	1.78	1.08–2.93	0.024
Non‐sarcopenia/short‐distance	3.20	1.81–5.67	<0.001	2.26	1.22–4.19	0.010
Sarcopenia/short‐distance	5.23	3.01–9.07	<0.001	3.38	1.79–6.37	<0.001
Age, per SD increase	1.22	0.99–1.50	0.068	1.32	1.03–1.69	0.026
Male	1.86	1.17–2.94	0.008	0.93	0.53–1.66	0.811
BMI, per SD decrease	1.28	1.05–1.56	0.013	1.20	0.97–1.49	0.098
Ever smoker	3.52	1.93–6.41	<0.001	2.35	1.10–5.01	0.027
Pathological stage						
0, I	1.00	[Reference]		1.00	[Reference]	
II	2.48	1.56–3.93	<0.001	2.29	1.39–3.78	0.001
III	3.54	2.25–5.57	<0.001	2.98	1.75–5.06	<0.001
Albumin, per SD decrease	1.55	1.35–1.79	<0.001	1.23	1.04–1.47	0.019
Sublobar resection	0.79	0.46–1.34	0.371	1.17	0.63–2.15	0.620
%FEV_1_, per SD decrease	1.31	1.08–1.59	<0.001	0.98	0.78–1.24	0.886
%Dlco, per SD decrease	1.79	1.45–2.20	<0.001	1.37	1.10–1.72	0.005
Disease‐free survival						
Indicator						
Non‐sarcopenia/long‐distance	1.00	[Reference]		1.00	[Reference]	
Sarcopenia/long‐distance	1.52	1.09–2.10	0.013	1.31	0.92–1.87	0.133
Non‐sarcopenia/short‐distance	2.44	1.62–3.65	<0.001	2.09	1.36–3.21	<0.001
Sarcopenia/short‐distance	2.32	1.47–3.66	<0.001	2.11	1.27–3.51	0.004
Age, per SD increase	1.10	0.95–1.27	0.215	1.18	1.00–1.38	0.044
Male	1.71	1.24–2.36	0.001	1.49	0.96–2.32	0.078
BMI, per SD decrease	1.10	0.96–1.26	0.172	1.11	0.95–1.30	0.172
Ever smoker	1.96	1.38–2.77	<0.001	1.17	0.73–1.89	0.509
Pathological stage						
0, I	1.00	[Reference]		1.00	[Reference]	
II	2.13	1.51–3.00	<0.001	1.95	1.35–2.81	<0.001
III	4.39	3.19–6.04	<0.001	4.30	2.99–6.20	<0.001
Albumin, per SD decrease	1.33	1.18–1.49	<0.001	1.04	0.91–1.19	0.525
Sublobar resection	0.70	0.47–1.03	0.071	1.00	0.64–1.55	0.994
%FEV_1_, per SD decrease	1.09	0.94–1.24	0.289	0.83	0.70–0.97	0.023
%Dlco, per SD decrease	1.37	1.19–1.59	<0.001	1.21	1.03–1.42	0.022

%DLco, percentage of predicted value of lung diffusion capacity for carbon monoxide; %FEV_1_, percentage of predicted value of forced expiratory volume in 1 s; 6MWD, 6 min walking distance; BMI, body mass index; CI, confidence interval; HR, hazard ratio; SD, standard deviation; SMI, skeletal muscle index.

Sarcopenia was defined as normalized SMI in the lowest sex‐specific tertile. Short‐distance was defined as 6MWD < 400 m.

**Figure 3:**
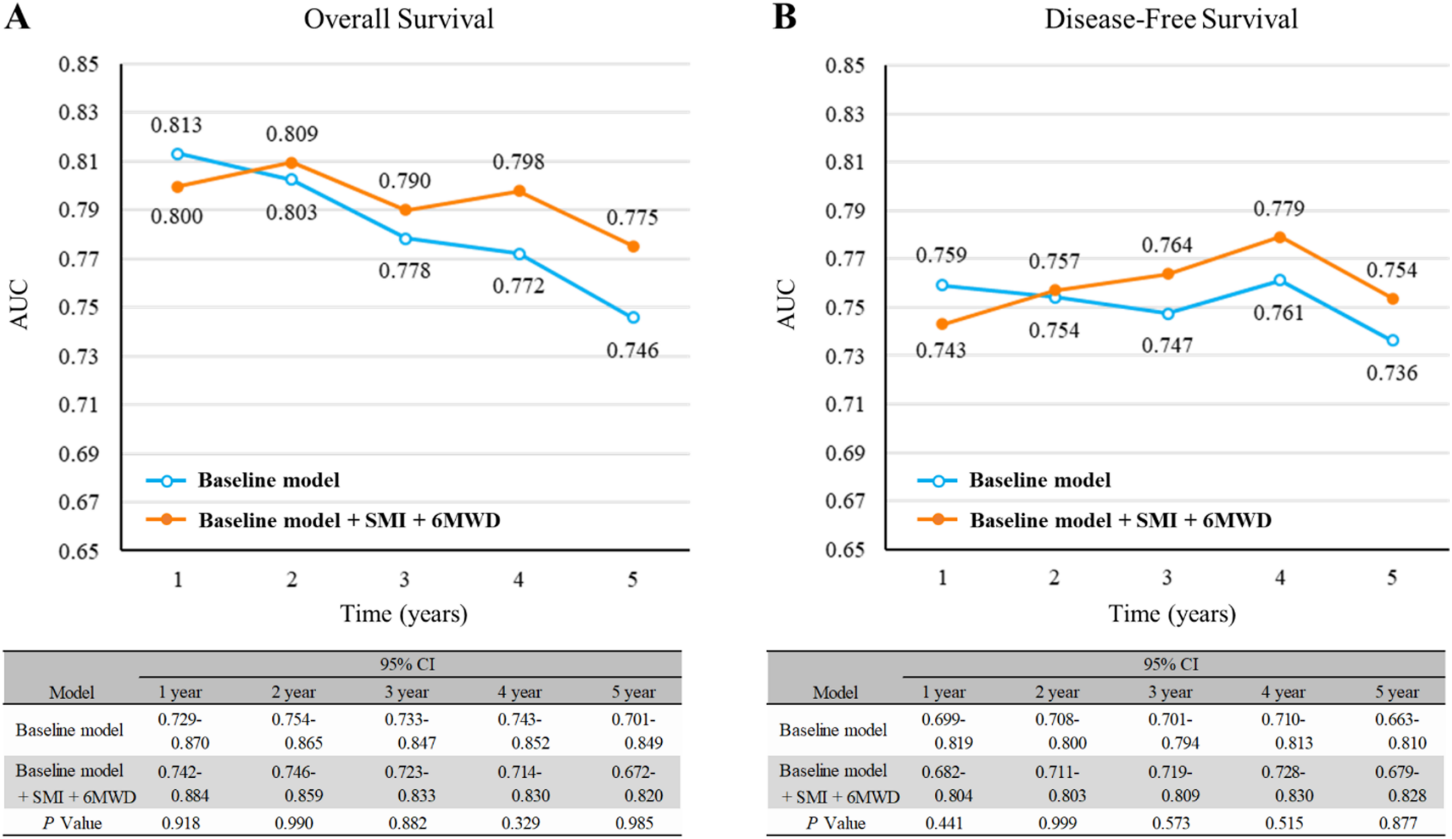
Plots of annual areas under the ROC curve (AUCs) for baseline model and baseline model plus skeletal muscle index (SMI) and 6 min walking distance (6MWD) for *(A)* overall survival and *(B)* disease‐free survival. The baseline model included age, sex, body mass index, smoking status, pathological stage, serum albumin, extent of resection, percentage of predicted value of forced expiratory volume in 1 s, and percentage of predicted value of lung diffusion capacity for carbon monoxide. CI, confidence interval.

Time‐dependent ROC analyses were performed for prediction of OS and DFS to evaluate the incremental prognostic predictive ability of preoperative SMI and 6MWD for each endpoint (*Figure* 3). The AUC for the baseline model was highest for OS in the short term but decreased with time after surgery. The AUCs for OS and DFS with addition of both SMI and 6MWD to the baseline model were higher, although the differences were not significant, than those for the baseline model, except within 2 years of surgery.

## Discussion

The present study was performed to investigate the prognostic significance of determination of preoperative paraspinous muscle sarcopenia at the T12 level by CT and of physical performance using the 6MWD in NSCLC. The results of the present study showed that BMI < 18.5 kg/m^2^ was associated with higher likelihoods of both sarcopenia and short distance in 6MWD. Both SMI and 6MWD were inversely related to OS. The groups of patients with both sarcopenia and short distance in 6MWD showed a significant association with longer post‐operative hospital stay and shorter OS and DFS. The combination of SMI and 6MWD had complementary prognostic predictive capability for pre‐existing prognostic factors, although this was not significant. These observations emphasize that a comprehensive evaluation taking into account both preoperative thoracic sarcopenia and physical performance is required in NSCLC.

Consistent with the results of the present study, preoperative sarcopenia has been reported to be an unfavourable prognostic factor for OS in NSCLC.^9^ Although a number of methods are available for assessment of skeletal muscle mass, the psoas muscle at the L3 level has been used in many studies as the reference for evaluation of sarcopenia by CT.^8^ Here, the paraspinous muscle area at the T12 level determined on chest CT was used to classify thoracic sarcopenia, which was also shown to be related to psoas muscle area at the L3 level,^13^ and to predict mortality in various populations including NSCLC patients.^11^, ^12^, ^13^, ^14^ It is recommended to obtain preoperative physical performance measurements for assessment of post‐operative outcome risks in NSCLC patients,^16^, ^27^ and the 6MWD is the most common such measurement.^17^, ^18^, ^28^ As an indicator of exercise capacity and walking endurance, the 6MWD has been shown to be closely related to quality of life, activities of daily living, mobility, hospitalization, and mortality in patients with respiratory disease.^29^ Consistent with our observations, preoperative 6MWD was shown to be inversely related with the risks of post‐operative cardiopulmonary complications and mortality in patients undergoing lung resection.^17^, ^18^, ^30^ The present study supported the use of routine clinical examination of preoperative sarcopenia and 6MWD in patients with NSCLC. When SMI and 6MWD were added to the prognostic factors, as the AUCs for OS and DFS tended to increase except within 2 years of surgery, these parameters may complement the ability to predict medium‐term to long‐term prognosis for existing prognostic factors. However, the 95% CI was wide and *P* value did not reach statistical significance. The small sample size, limited number of clinical events, and corrections for multiple comparisons may have reduced the statistical power to detect significance, and further studies in larger cohorts are therefore required.

In the present study, patients with both thoracic sarcopenia and short‐distance 6MWD had longer hospital stay and poorer prognosis with regard to OS and DFS. The combination of muscle loss and 6MWD < 400 m is defined as ‘sarcopenia with limited mobility’,^23^ which is included within the concept of frailty.^31^ Muscle mass is inversely associated with circulating inflammatory marker levels,^32^, ^33^ and sarcopenia has been shown to be related to elevated post‐operative inflammatory response.^34^ Physical performance and mobility are closely related to physical activity,^35^, ^36^ and physical activity has been suggested to counteract some specific hallmarks of cancer and to prevent chemotherapy‐related adverse events.^37^ In addition, the anti‐tumorigenic effect of exercise is related to immunomodulation, particularly by increasing pro‐inflammatory cytokine levels and natural killer cell infiltration in the tumour micro‐environment.^38^ Patients with low physical performance may not benefit from these effects, and therefore, these associations may be related to the high incidence of post‐operative adverse events in patients with ‘sarcopenia with limited mobility’. Moreover, the consensus of the European Working Group of Sarcopenia also defines persons have sarcopenia and cannot walk 400 m within 6 min as having ‘severe sarcopenia’.^7^ Severe sarcopenia has been shown to increase disability risk and mortality among community‐dwelling older adults.^39^, ^40^ In the present study, patients with BMI < 18.5 kg/m^2^ were more likely to be positive for severe sarcopenia. Cachexia is a generalized wasting process affecting all body compartments and is associated particularly with cancer where the prevalence can reach 50–80% in advanced malignant cancer and accounts death for up to 20% of cancer deaths.^41^ Cachexia is closely linked to skeletal muscle wasting associated with severe sarcopenia, and it has multifactorial causes characterized by catabolic/anabolic imbalance.^42^

The results of the present study have implications for both clinical practice and the design of future clinical studies regarding NSCLC. Accurate risk stratification is a prerequisite for the planning of medical care and improvement of prognosis of vulnerable populations. It is important to accurately assess preoperative sarcopenia and physical performance because both can be targeted for treatment before and after surgery with various interventions, including nutritional recommendations and rehabilitation.^43^, ^44^, ^45^ Exercise is a non‐pharmacological intervention that improves fatigue, quality of life, pulmonary function, muscle mass, physical performance, and psychological status of patients with lung cancer.^37^, ^38^ In addition, the performance of resistance training and use of nutritional supplements, including branched‐chain amino acids, vitamin D, whey protein, and hydroxymethylbutyrate‐enriched milk, play important roles in the prevention and improvement of sarcopenia,^15^ and these interventions may result in improved post‐operative outcomes for frail NSCLC patients. However, the effects of these interventions on thoracic sarcopenia are unknown, and it is unclear whether improvements in thoracic sarcopenia and low physical performance can improve prognosis. Therefore, further studies addressing these issues are required to facilitate future clinical decision making regarding the treatment of NSCLC.

This study had several limitations. First, this was a retrospective, single‐centre study in a small population with a short follow‐up period. Second, the study population included only Asian NSCLC patients, and preoperative sarcopenia was defined as the presence of normalized SMI in the lowest sex‐specific tertile with no other definitions of sarcopenia used in the evaluation. The original definition of sarcopenia by the consensus of the European Working Group on Sarcopenia added muscle function to the previous definitions based only on detection of low muscle mass, and the revised consensus focused on low muscle function, such as grip strength, as the primary parameter of sarcopenia. A recent study showed that patients with NSCLC who had a combination of handgrip weakness and low muscle mass, that is, actual sarcopenia, had a poor prognosis.^46^ The optimal assessment and cut‐off points for characterization of sarcopenia in NSCLS patients remain to be determined. Further studies are needed to validate thoracic sarcopenia in other populations and in larger cohorts, as well as comparisons with standard definitions of sarcopenia. Finally, although multivariate analysis can mitigate bias after adjusting for pre‐existing prognostic factors, other factors that were not measured and were unadjusted, such as physical activity and changes in baseline variables, can leave residual bias, and future studies are required to confirm our results.

In conclusion, preoperative paraspinous muscle sarcopenia and poor physical performance, as indicated by short‐distance 6MWD (<400 m), were shown to be associated with mortality rate of NSCLC patients, and the coexistence of both conditions had an adverse effect on prognosis in these patients. The results presented here suggested that preoperative thoracic sarcopenia and physical performance would be useful for accurate risk stratification of surgical candidates in NSCLC patients.

## Funding

This work was supported by the Japan Society for the Promotion of Science Grant‐in‐Aid (JSPS KAKENHI, Grant No. 20K19375).

## Conflict of interest

None declared.

## Supporting information


**Figure S1.** SMI and 6MWD in Subgroups

**Table S1.** Baseline Patient Characteristics According to the Sarcopenic Status and Physical Performance

**Table S2.** Adjusted Cox Regression Analyses for Postoperative Outcomes

**Table S3.** Sensitivity Analysis for Alternative Cutoff Values of SMI for Sarcopenia
