# Supplementary material for: Preoperative paraspinous muscle sarcopenia and physical performance as prognostic indicators in non‐small‐cell lung cancer
Source: J Cachexia Sarcopenia Muscle. 2021 Mar 4;12(3):646–56. doi: 10.1002/jcsm.12691 (PMC8200441; doi:10.1002/jcsm.12691)
Supplement: Supplementary file 1 — Figure S1. SMI and 6MWD in Subgroups [file JCSM-12-646-s001.docx]

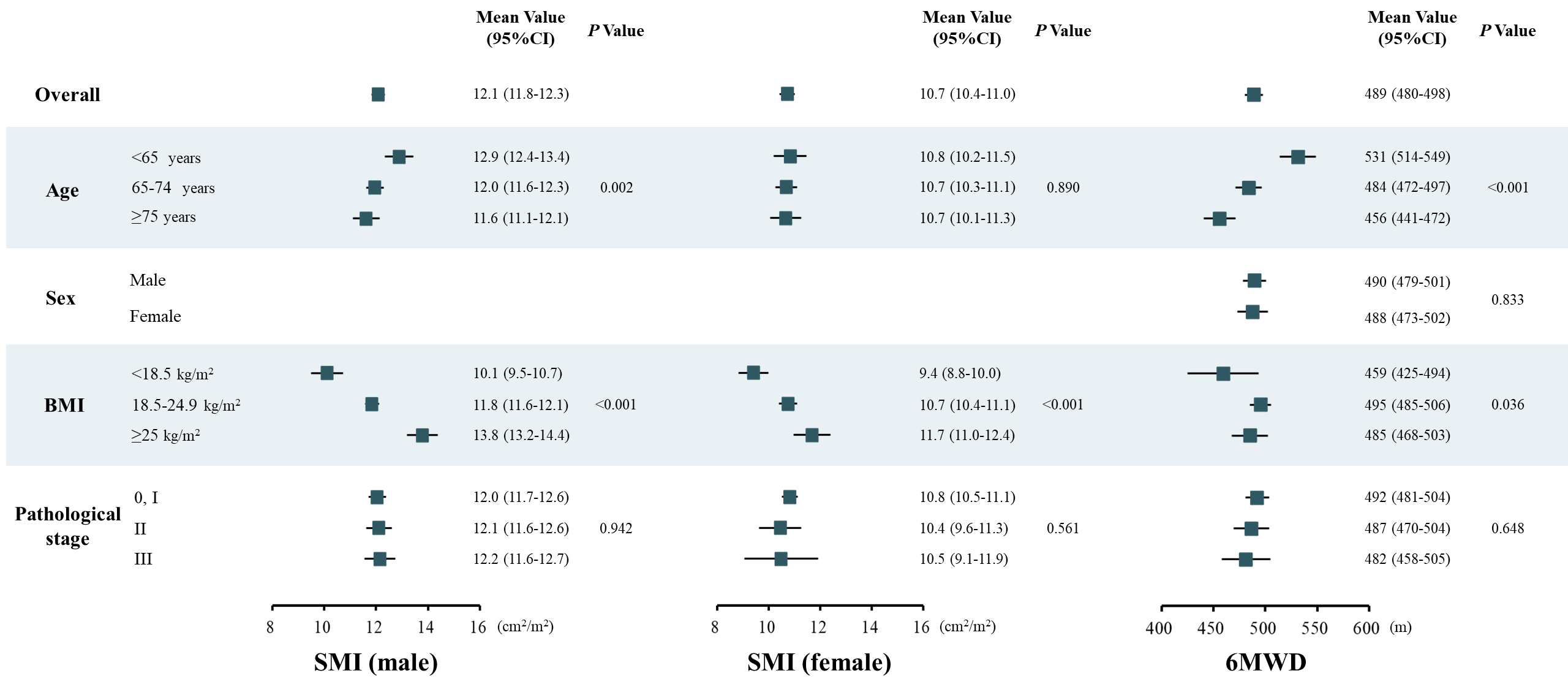


**Figure S1. SMI and 6MWD in Subgroups**

BMI, body mass index; CI, confidence interval; SMI, skeletal muscle index; 6MWD, 6-minute walking distance.
