# Supplementary material for: Preoperative paraspinous muscle sarcopenia and physical performance as prognostic indicators in non‐small‐cell lung cancer
Source: J Cachexia Sarcopenia Muscle. 2021 Mar 4;12(3):646–56. doi: 10.1002/jcsm.12691 (PMC8200441; doi:10.1002/jcsm.12691)
Supplement: Supplementary file 2 — Table S1. Baseline Patient Characteristics According to the Sarcopenic Status and Physical Performance [file JCSM-12-646-s003.docx]

| **Table S1. Baseline Patient Characteristics According to the Sarcopenic Status and Physical Performance** | | | | | | | | | | | | | | | | | | | |
| --- | --- | --- | --- | --- | --- | --- | --- | --- | --- | --- | --- | --- | --- | --- | --- | --- | --- | --- | --- |
|  |  | Non-sarcopenia | | |  | Sarcopenia | | | *P* Value |  | Long-distance | | |  | Short-distance | | | *P* Value |  |
|  |  | (n = 393; 67%) | | |  | (n = 194; 33%) | | |  |  | (n = 494; 84%) | | |  | (n = 93; 16%) | | |  |  |
| Age, mean (SD), y | | 68.2 | ± | 8.5 |  | 69.1 | ± | 9.3 | 0.244 |  | 68.0 | ± | 8.8 |  | 71.3 | ± | 7.8 | 0.001 |  |
| Age group, No. (%) | |  |  |  |  |  |  |  | 0.215 |  |  |  |  |  |  |  |  | <0.001 |  |
|  | <65 | 102 (26.0) | | |  | 38 (19.6) | | |  |  | 131 (26.5) | | |  | 9 (9.7) | | |  |  |
|  | 65-74 | 205 (52.2) | | |  | 107 (55.2) | | |  |  | 260 (52.6) | | |  | 52 (55.9) | | |  |  |
|  | ≥75 | 86 (21.9) | | |  | 49 (25.3) | | |  |  | 103 (20.9) | | |  | 32 (34.4) | | |  |  |
| Male, No. (%) | | 267 (67.9) | | |  | 132 (68.0) | | | 1.000 |  | 333 (67.4) | | |  | 66 (71.0) | | | 0.546 |  |
| Height, mean (SD), cm | | 161.1 | ± | 8.6 |  | 161.1 | ± | 8.6 | 0.927 |  | 161.4 | ± | 8.5 |  | 159.4 | ± | 8.8 | 0.035 |  |
| Body weight, mean (SD), kg | | 60.3 | ± | 10.5 |  | 54.5 | ± | 10.2 | <0.001 |  | 58.7 | ± | 10.8 |  | 56.4 | ± | 10.6 | 0.060 |  |
| BMI, mean (SD), kg/m^2^ | | 23.1 | ± | 3.1 |  | 20.9 | ± | 3.2 | <0.001 |  | 22.5 | ± | 3.3 |  | 22.2 | ± | 3.6 | 0.462 |  |
| BMI group, No. (%) | |  |  |  |  |  |  |  | <0.001 |  |  |  |  |  |  |  |  | 0.064 |  |
|  | <18.5 | 22 (5.6) | | |  | 48 (24.7) | | |  |  | 52 (10.5) | | |  | 18 (19.4) | | |  |  |
|  | 18.5-24.9 | 269 (68.4) | | |  | 122 (62.9) | | |  |  | 335 (67.8) | | |  | 56 (60.2) | | |  |  |
|  | ≥25 | 102 (26.0) | | |  | 24 (12.4) | | |  |  | 107 (21.7) | | |  | 19 (20.4) | | |  |  |
| Smoking status, No. (%) | |  | | |  |  | | | 1.000 |  |  | | |  |  | | | 0.105 |  |
|  | Never smoker | 113 (28.8) | | |  | 55 (28.4) | | |  |  | 148 (30.0) | | |  | 20 (21.5) | | |  |  |
|  | Ever smoker (current or ex-smoker) | 280 (71.2) | | |  | 139 (71.6) | | |  |  | 346 (70.0) | | |  | 73 (78.5) | | |  |  |
| Comorbidities, No. (%) | |  |  |  |  |  |  |  |  |  |  |  |  |  |  |  |  |  |  |
|  | COPD | 148 (37.7) | | |  | 72 (37.1) | | | 0.928 |  | 189 (38.3) | | |  | 31 (33.3) | | | 0.414 |  |
|  | Diabetes | 88 (22.4) | | |  | 47 (24.2) | | | 0.677 |  | 106 (21.5) | | |  | 29 (31.2) | | | 0.045 |  |
|  | Hypertension | 135 (34.4) | | |  | 68 (35.1) | | | 0.927 |  | 165 (33.4) | | |  | 38 (40.9) | | | 0.191 |  |
|  | Chronic kidney disease | 94 (23.9) | | |  | 37 (19.1) | | | 0.207 |  | 105 (21.3) | | |  | 26 (28.0) | | | 0.174 |  |
|  | Anemia | 113 (28.8) | | |  | 86 (44.3) | | | <0.001 |  | 151 (30.6) | | |  | 48 (51.6) | | | <0.001 |  |
|  | **Peripheral vascular disease** | **14 (3.6)** | | |  | **11 (5.7)** | | | **0.277** |  | **8 (1.6)** | | |  | **17 (18.3)** | | | **<0.001** |  |
|  | **Cerebrovascular disease** | **26 (6.6)** | | |  | **12 (6.2)** | | | **1.000** |  | **27 (5.5)** | | |  | **11 (11.8)** | | | **0.035** |  |
| Laboratory findings | |  |  |  |  |  |  |  |  |  |  |  |  |  |  |  |  |  |  |
|  | Albumin, mean (SD), g/dL | 4.0 | ± | 0.4 |  | 3.9 | ± | 0.5 | 0.001 |  | 4.0 | ± | 0.4 |  | 3.9 | ± | 0.5 | 0.002 |  |
|  | Hemoglobin, mean (SD), g/dL | 13.2 | ± | 1.5 |  | 12.7 | ± | 1.7 | <0.001 |  | 13.1 | ± | 1.6 |  | 12.6 | ± | 1.6 | 0.002 |  |
|  | Creatinine, mean (SD), mg/dL | 0.93 | ± | 1.05 |  | 0.85 | ± | 0.68 | 0.290 |  | 0.87 | ± | 0.82 |  | 1.11 | ± | 1.42 | 0.025 |  |
|  | eGFR, mean (SD), mL/min/1.73 m^2^ | 70.6 | ± | 18.9 |  | 76.7 | ± | 26.2 | 0.002 |  | 72.6 | ± | 19.0 |  | 72.7 | ± | 32.8 | 0.976 |  |
|  | CRP, median (IQR), mg/L | 0.08 | [0.03 - 0.24] | |  | 0.09 | [0.04 - 0.27] | | 0.665 |  | 0.07 | [0.03 - 0.24] | |  | 0.12 | [0.07 - 0.28] | | 0.001 |  |
|  | TLC, mean (SD), cells/mm^3^ | 1741 | ± | 630 |  | 1649 | ± | 577 | 0.087 |  | 1719 | ± | 619 |  | 1665 | ± | 592 | 0.429 |  |
|  | TNC, mean (SD), cells/mm^3^ | 4241 | ± | 1548 |  | 4634 | ± | 3023 | 0.038 |  | 4301 | ± | 1733 |  | 4742 | ± | 3652 | 0.070 |  |
|  | NLR, mean (SD) | 2.8 | ± | 2.0 |  | 3.2 | ± | 2.4 | 0.080 |  | 2.9 | ± | 2.1 |  | 3.2 | ± | 2.2 | 0.154 |  |
| Pathological stage, No. (%) | |  |  |  |  |  |  |  | 0.746 |  |  |  |  |  |  |  |  | 0.664 |  |
|  | 0, I | 234 (59.5) | | |  | 110 (56.7) | | |  |  | 292 (59.1) | | |  | 52 (55.9) | | |  |  |
|  | II | 87 (22.1) | | |  | 44 (22.7) | | |  |  | 107 (21.7) | | |  | 24 (25.8) | | |  |  |
|  | III | 72 (18.3) | | |  | 40 (20.6) | | |  |  | 95 (19.2) | | |  | 17 (18.3) | | |  |  |
| Histologic type, No. (%) | |  |  |  |  |  |  |  | 0.550 |  |  |  |  |  |  |  |  | 0.265 |  |
|  | Squamous cell carcinoma | 107 (27.2) | | |  | 60 (30.9) | | |  |  | 134 (27.1) | | |  | 33 (35.5) | | |  |  |
|  | Adenocarcinoma | 266 (67.7) | | |  | 127 (65.5) | | |  |  | 337 (68.2) | | |  | 56 (60.2) | | |  |  |
|  | Others | 20 (5.1) | | |  | 7 (3.6) | | |  |  | 23 (4.7) | | |  | 4 (4.3) | | |  |  |
| Operation time, median (IQR), min | | 149 | [117 - 185] | |  | 155 | [126 - 189] | | 0.171 |  | 151 | [120 - 187] | |  | 145 | [116 - 176] | | 0.200 |  |
| Blood loss during surgery, median (IQR), g | | 46 | [17 - 130] | |  | 66 | [24 - 186] | | 0.046 |  | 51 | [19 - 139] | |  | 61 | [18 - 184] | | 0.612 |  |
| **Surgical approach, n (%)** | |  |  |  |  |  |  |  | **0.093** |  |  |  |  |  |  |  |  | **0.820** |  |
|  | **Open** | **163 (41.5)** | | |  | **95 (49.0)** | | |  |  | **216 (43.7)** | | |  | **42 (45.2)** | | |  |  |
|  | **VATS** | **230 (58.5)** | | |  | **99 (51.0)** | | |  |  | **278 (56.3)** | | |  | **51 (54.8)** | | |  |  |
| **Extent of resection, n (%)** | |  | | |  |  | | | **0.083** |  |  | | |  |  | | | **1.000** |  |
|  | **Lobectomy** | **317 (80.7)** | | |  | **168 (86.6)** | | |  |  | **408 (82.6)** | | |  | **77 (82.8)** | | |  |  |
|  | **Sublobar resection** | **76 (19.3)** | | |  | **26 (13.4)** | | |  |  | **86 (17.4)** | | |  | **16 (17.2)** | | |  |  |
| **Neoadjuvant therapy, n (%)** | | **17 (4.3)** | | |  | **7 (3.6)** | | | **0.826** |  | **20 (4.0)** | | |  | **4 (4.3)** | | | **0.782** |  |
| **Adjuvant therapy, n (%)** | | **38 (9.7)** | | |  | **11 (5.7)** | | | **0.114** |  | **43 (8.7)** | | |  | **6 (6.5)** | | | **0.547** |  |
| Preoperative pulmonary function test | |  |  |  |  |  |  |  |  |  |  |  |  |  |  |  |  |  |  |
|  | VC, mean (SD), L | 3.36 | ± | 0.78 |  | 3.1 | ± | 0.76 | <0.001 |  | 3.34 | ± | 0.77 |  | 2.93 | ± | 0.76 | <0.001 |  |
|  | VC, mean (SD), %predicted | 104.2 | ± | 14.99 |  | 97.57 | ± | 17.55 | <0.001 |  | 103.3 | ± | 15.42 |  | 95.28 | ± | 18.35 | <0.001 |  |
|  | FEV_1_, mean (SD), L | 2.32 | ± | 0.58 |  | 2.19 | ± | 0.57 | 0.010 |  | 2.32 | ± | 0.58 |  | 2.04 | ± | 0.56 | <0.001 |  |
|  | FEV_1_, mean (SD), %predicted | 93.51 | ± | 18.39 |  | 90.24 | ± | 20.89 | 0.054 |  | 93.35 | ± | 18.87 |  | 87.53 | ± | 20.85 | 0.008 |  |
|  | FEV_1_/FVC, mean (SD) | 69.43 | ± | 9.27 |  | 71.24 | ± | 11.48 | 0.041 |  | 69.96 | ± | 9.64 |  | 70.4 | ± | 12.22 | 0.697 |  |
|  | DLco, mean (SD), %predicted | 104 | ± | 25.13 |  | 101.1 | ± | 27.34 | 0.208 |  | 104.6 | ± | 25.08 |  | 94.57 | ± | 28.58 | 0.001 |  |
| SMI, mean (SD), cm^2^/m^2^ | |  |  |  |  |  |  |  |  |  |  |  |  |  |  |  |  |  |  |
|  | male | 13.4 | ± | 1.8 |  | 9.4 | ± | 1.6 | <0.001 |  | 12.3 | ± | 2.6 |  | 11.2 | ± | 2.5 | 0.001 |  |
|  | female | 11.8 | ± | 1.5 |  | 8.6 | ± | 1.4 | <0.001 |  | 10.8 | ± | 2.0 |  | 10.2 | ± | 2.6 | 0.193 |  |
| 6MWD, mean (SD), m | | 499 | ± | 100 |  | 469 | ± | 123 | 0.002 |  | 523 | ± | 74 |  | 310 | ± | 88 | <0.001 |  |
| BMI, body mass index; COPD, chronic obstructive pulmonary disease; CRP, C-reactive protein; DLco, lung diffusion capacity for carbon monoxide; eGFR, estimated glomerular filtration rate; FEV1, forced expiratory volume in 1 second; FVC, forced vital capacity; IQR, interquartile range; NLR, neutrophil lymphocyte ratio; SMI, skeletal muscle index; TLC, total lymphocyte count; TNC, total neutrophil count; VATS, video-assisted thoracic surgery; VC, vital capacity; 6MWD, 6-minute walking distance.  Sarcopenia was defined as normalized SMI in the lowest sex-specific tertile.  Short-distance was defined as 6MWD < 400 m. | | | | | | | | | | | | | | | | | | |  |
