# Supplementary material for: Preoperative paraspinous muscle sarcopenia and physical performance as prognostic indicators in non‐small‐cell lung cancer
Source: J Cachexia Sarcopenia Muscle. 2021 Mar 4;12(3):646–56. doi: 10.1002/jcsm.12691 (PMC8200441; doi:10.1002/jcsm.12691)
Supplement: Supplementary file 3 — Table S2. Adjusted Cox Regression Analyses for Postoperative Outcomes [file JCSM-12-646-s002.docx]

| **Table S2. Adjusted Cox Regression Analyses for Postoperative Outcomes** | | | | | | | | | | |
| --- | --- | --- | --- | --- | --- | --- | --- | --- | --- | --- |
|  |  |  | Overall survival | | |  | Disease-free survival | | |  |
|  | Variable | | HR | 95% CI | *P* Value |  | HR | 95% CI | *P* Value |  |
|  | SMI, per 1 cm^2^/m^2^ decrease | | 1.09 | 1.01-1.18 | 0.025 |  | 1.05 | 0.99-1.11 | 0.099 |  |
|  | Sarcopenia (vs non-sarcopenia) | | 1.75 | 1.14-2.67 | 0.010 |  | 1.26 | 0.92-1.72 | 0.145 |  |
|  | 6MWD, per 50 m decrease | | 1.14 | 1.05-1.25 | 0.003 |  | 1.09 | 1.02-1.17 | 0.008 |  |
|  | Short-distance (vs long-distance) | | 2.13 | 1.37-3.32 | <0.001 |  | 1.89 | 1.35-2.65 | <0.001 |  |
| BMI, body mass index; CI, confidence interval; HR, hazard ratio; %DLco, percent of predicted value of diffusion capacity of the lung for carbon monoxide; **%FEV1, percentage of predicted value of forced expiratory volume in 1 second**; SMI, skeletal muscle index; 6MWD, 6-minute walk distance. Data are presented as adjusted hazard ratio. Adjusted for age, sex, BMI, smoking status, pathological stage, serum albumin, **extent of resection, %FEV1,** and %DLco. Defined as sarcopenia, the presence of normalized SMI in the lowest sex-specific tertile. Defined as short-distance, the presence of 6MWD < 400 m. | | | | | | | | | | |
