# Supplementary material for: Preoperative paraspinous muscle sarcopenia and physical performance as prognostic indicators in non‐small‐cell lung cancer
Source: J Cachexia Sarcopenia Muscle. 2021 Mar 4;12(3):646–56. doi: 10.1002/jcsm.12691 (PMC8200441; doi:10.1002/jcsm.12691)
Supplement: Supplementary file 4 — Table S3. Sensitivity Analysis for Alternative Cutoff Values of SMI for Sarcopenia [file JCSM-12-646-s004.docx]

| **Table S3. Sensitivity Analysis for Alternative Cutoff Values of SMI for Sarcopenia** | | | | | | | | | | | | |
| --- | --- | --- | --- | --- | --- | --- | --- | --- | --- | --- | --- | --- |
|  |  |  | **Total number** | **Overall survival** | | |  | **Disease-free survival** | | |  |  |
|  | **Variable** | |  | **Adjusted HR** | **95% CI** | ***P* Value** |  | **Adjusted HR** | **95% CI** | ***P* Value** |  |  |
|  | **Cutoff: lowest sex-specific quartile** | |  |  |  |  |  |  |  |  |  |  |
|  |  | **Male < 10.58 cm^2^/m^2^, Female < 9.49 cm^2^/m^2^** |  |  |  |  |  |  |  |  |  |  |
|  |  | **Sarcopenia (vs. non-sarcopenia)** | **147** | **1.59** | **1.03-2.45** | **0.036** |  | **1.26** | **0.91-1.75** | **0.157** |  |  |
|  | **Cutoff: Kaplan's value^13^** | |  |  |  |  |  |  |  |  |  |  |
|  |  | **Male < 10.66 cm^2^/m^2^, Female < 9.01 cm^2^/m^2^** |  |  |  |  |  |  |  |  |  |  |
|  |  | **Sarcopenia (vs. non-sarcopenia)** | **141** | **1.57** | **1.02-2.41** | **0.039** |  | **1.38** | **1.00-1.90** | **0.050** |  |  |
| **BMI, body mass index; CI, confidence interval; HR, hazard ratio; %DLco, percentage of predicted value of lung diffusion capacity for carbon monoxide; %FEV1, percentage of predicted value of forced expiratory volume in 1 second; SMI, skeletal muscle index. Adjusted for age, sex, BMI, smoking status, pathological stage, serum albumin, extent of resection, %FEV1, and %DLco.** | | | | | | | | | | | | |
